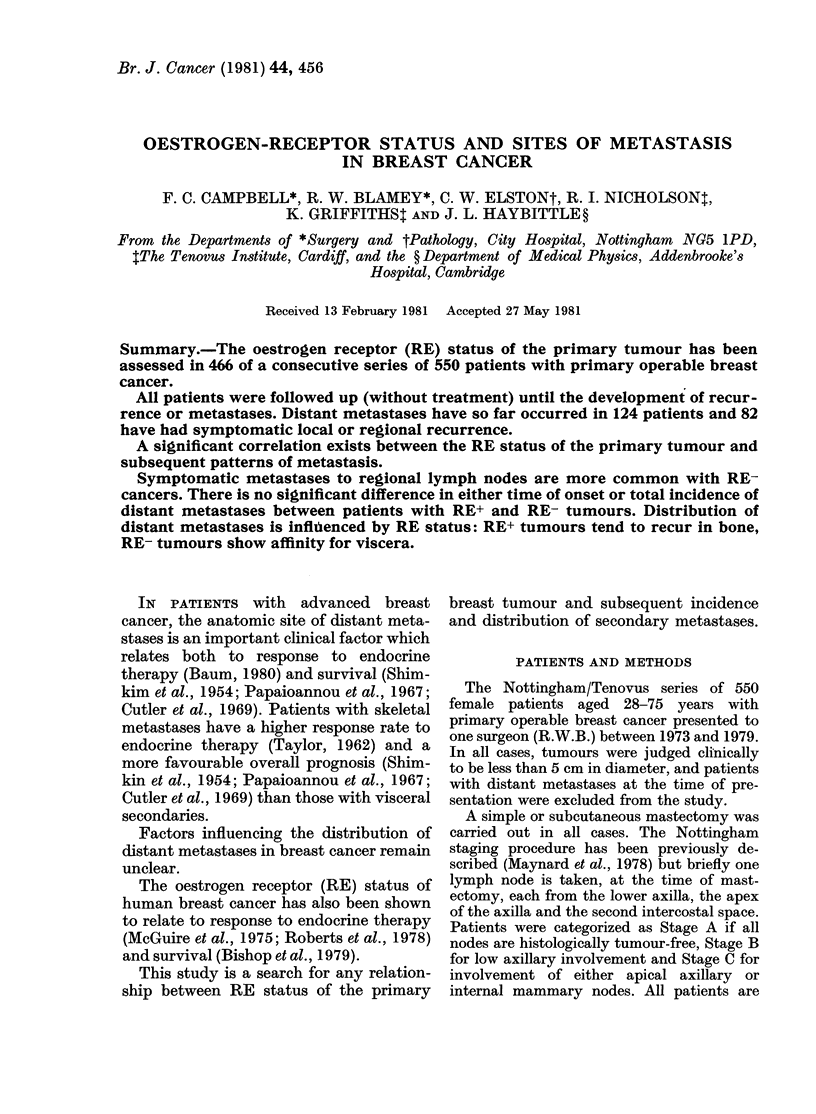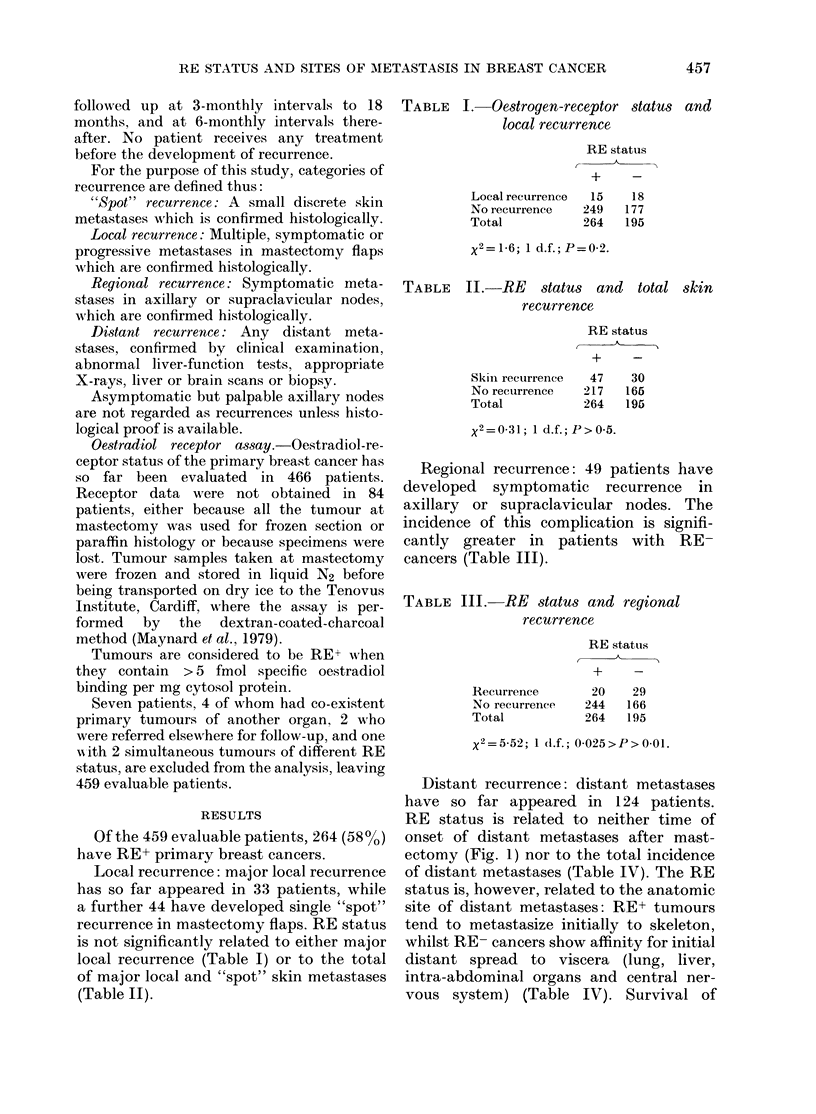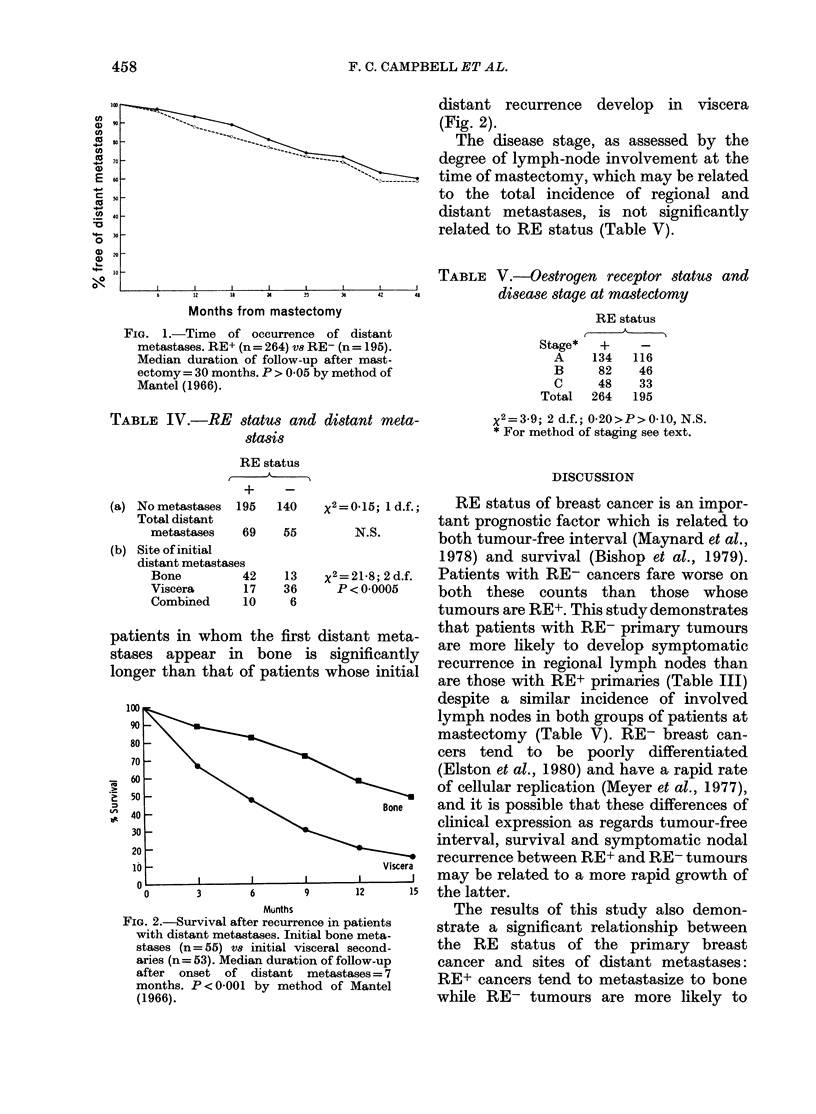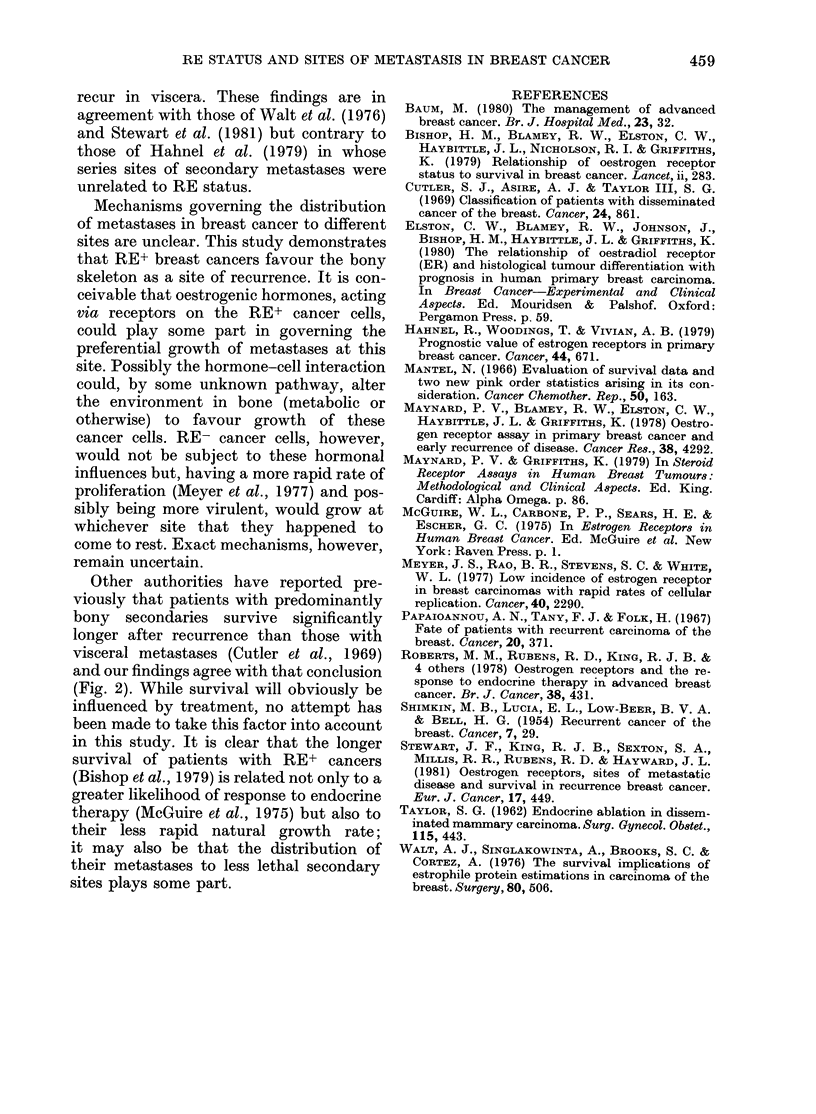# Oestrogen-receptor status and sites of metastasis in breast cancer.

**DOI:** 10.1038/bjc.1981.205

**Published:** 1981-09

**Authors:** F. C. Campbell, R. W. Blamey, C. W. Elston, R. I. Nicholson, K. Griffiths, J. L. Haybittle

## Abstract

The oestrogen receptor (RE) status of the primary tumour has been assessed in 466 of a consecutive series of 550 patients with primary operable breast cancer. All patients were followed up (without treatment) until the development of recurrence or metastases. Distant metastases have so far occurred in 124 patients and 82 have had symptomatic local or regional recurrence. A significant correlation exists between the RE status of the primary tumour and subsequent patterns of metastasis. Symptomatic metastases to regional lymph nodes are more common with RE- cancers. There is no significant difference in either time of onset or total incidence of distant metastases between patients with RE+ and RE- tumours. Distribution of distant metastases is influenced by RE status: RE+ tumours tend to recur in bone, RE- tumours show affinity for viscera.


*Br. J. Cancer* (1981) **44**, 456

### OESTROGEN-RECEPTOR STATUS AND SITES OF METASTASIS IN BREAST CANCER

F. C. CAMPBELL*, R. W. BLAMEY*, C. W. ELSTON†, R. I. NICHOLSON‡, K. GRIFFITHS‡ AND J. L. HAYBITTLE§

*From the Departments of *Surgery and †Pathology, City Hospital, Nottingham NG5 1PD, ‡The Tenovus Institute, Cardiff, and the § Department of Medical Physics, Addenbrooke's Hospital, Cambridge*

Received 13 February 1981 Accepted 27 May 1981

**Summary.—The oestrogen receptor (RE) status of the primary tumour has been assessed in 466 of a consecutive series of 550 patients with primary operable breast cancer.**

**All patients were followed up (without treatment) until the development of recurrence or metastases. Distant metastases have so far occurred in 124 patients and 82 have had symptomatic local or regional recurrence.**

**A significant correlation exists between the RE status of the primary tumour and subsequent patterns of metastasis.**

**Symptomatic metastases to regional lymph nodes are more common with RE$^-$ cancers. There is no significant difference in either time of onset or total incidence of distant metastases between patients with RE$^+$ and RE$^-$ tumours. Distribution of distant metastases is influenced by RE status: RE$^+$ tumours tend to recur in bone, RE$^-$ tumours show affinity for viscera.**

IN PATIENTS with advanced breast cancer, the anatomic site of distant metastases is an important clinical factor which relates both to response to endocrine therapy (Baum, 1980) and survival (Shimkim *et al.*, 1954; Papaioannou *et al.*, 1967; Cutler *et al.*, 1969). Patients with skeletal metastases have a higher response rate to endocrine therapy (Taylor, 1962) and a more favourable overall prognosis (Shimkin *et al.*, 1954; Papaioannou *et al.*, 1967; Cutler *et al.*, 1969) than those with visceral secondaries.

Factors influencing the distribution of distant metastases in breast cancer remain unclear.

The oestrogen receptor (RE) status of human breast cancer has also been shown to relate to response to endocrine therapy (McGuire *et al.*, 1975; Roberts *et al.*, 1978) and survival (Bishop *et al.*, 1979).

This study is a search for any relationship between RE status of the primary breast tumour and subsequent incidence and distribution of secondary metastases.

### PATIENTS AND METHODS

The Nottingham/Tenovus series of 550 female patients aged 28–75 years with primary operable breast cancer presented to one surgeon (R.W.B.) between 1973 and 1979. In all cases, tumours were judged clinically to be less than 5 cm in diameter, and patients with distant metastases at the time of presentation were excluded from the study.

A simple or subcutaneous mastectomy was carried out in all cases. The Nottingham staging procedure has been previously described (Maynard *et al.*, 1978) but briefly one lymph node is taken, at the time of mastectomy, each from the lower axilla, the apex of the axilla and the second intercostal space. Patients were categorized as Stage A if all nodes are histologically tumour-free, Stage B for low axillary involvement and Stage C for involvement of either apical axillary or internal mammary nodes. All patients are followed up at 3-monthly intervals to 18 months, and at 6-monthly intervals thereafter. No patient receives any treatment before the development of recurrence.

For the purpose of this study, categories of recurrence are defined thus:

*"Spot" recurrence:* A small discrete skin metastases which is confirmed histologically.

*Local recurrence:* Multiple, symptomatic or progressive metastases in mastectomy flaps which are confirmed histologically.

*Regional recurrence:* Symptomatic metastases in axillary or supraclavicular nodes, which are confirmed histologically.

*Distant recurrence:* Any distant metastases, confirmed by clinical examination, abnormal liver-function tests, appropriate X-rays, liver or brain scans or biopsy.

Asymptomatic but palpable axillary nodes are not regarded as recurrences unless histological proof is available.

*Oestradiol receptor assay.*—Oestradiol-receptor status of the primary breast cancer has so far been evaluated in 466 patients. Receptor data were not obtained in 84 patients, either because all the tumour at mastectomy was used for frozen section or paraffin histology or because specimens were lost. Tumour samples taken at mastectomy were frozen and stored in liquid $N_2$ before being transported on dry ice to the Tenovus Institute, Cardiff, where the assay is performed by the dextran-coated-charcoal method (Maynard *et al.*, 1979).

Tumours are considered to be $RE^+$ when they contain >5 fmol specific oestradiol binding per mg cytosol protein.

Seven patients, 4 of whom had co-existent primary tumours of another organ, 2 who were referred elsewhere for follow-up, and one with 2 simultaneous tumours of different RE status, are excluded from the analysis, leaving 459 evaluable patients.

### RESULTS

Of the 459 evaluable patients, 264 (58%) have $RE^+$ primary breast cancers.

Local recurrence: major local recurrence has so far appeared in 33 patients, while a further 44 have developed single "spot" recurrence in mastectomy flaps. RE status is not significantly related to either major local recurrence (Table I) or to the total of major local and "spot" skin metastases (Table II).

TABLE I.—*Oestrogen-receptor status and local recurrence*

| | RE status | |
|---|---|---|
| | + | − |
| Local recurrence | 15 | 18 |
| No recurrence | 249 | 177 |
| Total | 264 | 195 |

$\chi^2 = 1{\cdot}6$; 1 d.f.; $P = 0{\cdot}2$.

TABLE II.—*RE status and total skin recurrence*

| | RE status | |
|---|---|---|
| | + | − |
| Skin recurrence | 47 | 30 |
| No recurrence | 217 | 165 |
| Total | 264 | 195 |

$\chi^2 = 0{\cdot}31$; 1 d.f.; $P > 0{\cdot}5$.

Regional recurrence: 49 patients have developed symptomatic recurrence in axillary or supraclavicular nodes. The incidence of this complication is significantly greater in patients with $RE^-$ cancers (Table III).

TABLE III.—*RE status and regional recurrence*

| | RE status | |
|---|---|---|
| | + | − |
| Recurrence | 20 | 29 |
| No recurrence | 244 | 166 |
| Total | 264 | 195 |

$\chi^2 = 5{\cdot}52$; 1 d.f.; $0{\cdot}025 > P > 0{\cdot}01$.

Distant recurrence: distant metastases have so far appeared in 124 patients. RE status is related to neither time of onset of distant metastases after mastectomy (Fig. 1) nor to the total incidence of distant metastases (Table IV). The RE status is, however, related to the anatomic site of distant metastases: $RE^+$ tumours tend to metastasize initially to skeleton, whilst $RE^-$ cancers show affinity for initial distant spread to viscera (lung, liver, intra-abdominal organs and central nervous system) (Table IV). Survival of

FIG. 1.—Time of occurrence of distant metastases. $RE^+$ (n = 264) *vs* $RE^-$ (n = 195). Median duration of follow-up after mastectomy = 30 months. $P > 0{\cdot}05$ by method of Mantel (1966).

TABLE IV.—*RE status and distant metastasis*

| | RE status + | RE status − | |
|---|---|---|---|
| (a) No metastases | 195 | 140 | $\chi^2 = 0{\cdot}15$; 1 d.f.; |
| Total distant metastases | 69 | 55 | N.S. |
| (b) Site of initial distant metastases | | | |
| Bone | 42 | 13 | $\chi^2 = 21{\cdot}8$; 2 d.f. |
| Viscera | 17 | 36 | $P < 0{\cdot}0005$ |
| Combined | 10 | 6 | |

patients in whom the first distant metastases appear in bone is significantly longer than that of patients whose initial

FIG. 2.—Survival after recurrence in patients with distant metastases. Initial bone metastases (n = 55) *vs* initial visceral secondaries (n = 53). Median duration of follow-up after onset of distant metastases = 7 months. $P < 0{\cdot}001$ by method of Mantel (1966).

distant recurrence develop in viscera (Fig. 2).

The disease stage, as assessed by the degree of lymph-node involvement at the time of mastectomy, which may be related to the total incidence of regional and distant metastases, is not significantly related to RE status (Table V).

TABLE V.—*Oestrogen receptor status and disease stage at mastectomy*

| Stage* | RE status + | RE status − |
|---|---|---|
| A | 134 | 116 |
| B | 82 | 46 |
| C | 48 | 33 |
| Total | 264 | 195 |

$\chi^2 = 3{\cdot}9$; 2 d.f.; $0{\cdot}20 > P > 0{\cdot}10$, N.S.
* For method of staging see text.

### DISCUSSION

RE status of breast cancer is an important prognostic factor which is related to both tumour-free interval (Maynard *et al.*, 1978) and survival (Bishop *et al.*, 1979). Patients with $RE^-$ cancers fare worse on both these counts than those whose tumours are $RE^+$. This study demonstrates that patients with $RE^-$ primary tumours are more likely to develop symptomatic recurrence in regional lymph nodes than are those with $RE^+$ primaries (Table III) despite a similar incidence of involved lymph nodes in both groups of patients at mastectomy (Table V). $RE^-$ breast cancers tend to be poorly differentiated (Elston *et al.*, 1980) and have a rapid rate of cellular replication (Meyer *et al.*, 1977), and it is possible that these differences of clinical expression as regards tumour-free interval, survival and symptomatic nodal recurrence between $RE^+$ and $RE^-$ tumours may be related to a more rapid growth of the latter.

The results of this study also demonstrate a significant relationship between the RE status of the primary breast cancer and sites of distant metastases: $RE^+$ cancers tend to metastasize to bone while $RE^-$ tumours are more likely to recur in viscera. These findings are in agreement with those of Walt *et al.* (1976) and Stewart *et al.* (1981) but contrary to those of Hahnel *et al.* (1979) in whose series sites of secondary metastases were unrelated to RE status.

Mechanisms governing the distribution of metastases in breast cancer to different sites are unclear. This study demonstrates that $RE^+$ breast cancers favour the bony skeleton as a site of recurrence. It is conceivable that oestrogenic hormones, acting *via* receptors on the $RE^+$ cancer cells, could play some part in governing the preferential growth of metastases at this site. Possibly the hormone–cell interaction could, by some unknown pathway, alter the environment in bone (metabolic or otherwise) to favour growth of these cancer cells. $RE^-$ cancer cells, however, would not be subject to these hormonal influences but, having a more rapid rate of proliferation (Meyer *et al.*, 1977) and possibly being more virulent, would grow at whichever site that they happened to come to rest. Exact mechanisms, however, remain uncertain.

Other authorities have reported previously that patients with predominantly bony secondaries survive significantly longer after recurrence than those with visceral metastases (Cutler *et al.*, 1969) and our findings agree with that conclusion (Fig. 2). While survival will obviously be influenced by treatment, no attempt has been made to take this factor into account in this study. It is clear that the longer survival of patients with $RE^+$ cancers (Bishop *et al.*, 1979) is related not only to a greater likelihood of response to endocrine therapy (McGuire *et al.*, 1975) but also to their less rapid natural growth rate; it may also be that the distribution of their metastases to less lethal secondary sites plays some part.

### REFERENCES

Baum, M. (1980) The management of advanced breast cancer. *Br. J. Hospital Med.*, **23**, 32.

Bishop, H. M., Blamey, R. W., Elston, C. W., Haybittle, J. L., Nicholson, R. I. & Griffiths, K. (1979) Relationship of oestrogen receptor status to survival in breast cancer. *Lancet*, ii, 283.

Cutler, S. J., Asire, A. J. & Taylor III, S. G. (1969) Classification of patients with disseminated cancer of the breast. *Cancer*, **24**, 861.

Elston, C. W., Blamey, R. W., Johnson, J., Bishop, H. M., Haybittle, J. L. & Griffiths, K. (1980) The relationship of oestradiol receptor (ER) and histological tumour differentiation with prognosis in human primary breast carcinoma. In *Breast Cancer—Experimental and Clinical Aspects*. Ed. Mouridsen & Palshof. Oxford: Pergamon Press. p. 59.

Hahnel, R., Woodings, T. & Vivian, A. B. (1979) Prognostic value of estrogen receptors in primary breast cancer. *Cancer*, **44**, 671.

Mantel, N. (1966) Evaluation of survival data and two new pink order statistics arising in its consideration. *Cancer Chemother. Rep.*, **50**, 163.

Maynard, P. V., Blamey, R. W., Elston, C. W., Haybittle, J. L. & Griffiths, K. (1978) Oestrogen receptor assay in primary breast cancer and early recurrence of disease. *Cancer Res.*, **38**, 4292.

Maynard, P. V. & Griffiths, K. (1979) In *Steroid Receptor Assays in Human Breast Tumours: Methodological and Clinical Aspects*. Ed. King. Cardiff: Alpha Omega. p. 86.

McGuire, W. L., Carbone, P. P., Sears, H. E. & Escher, G. C. (1975) In *Estrogen Receptors in Human Breast Cancer*. Ed. McGuire *et al.* New York: Raven Press. p. 1.

Meyer, J. S., Rao, B. R., Stevens, S. C. & White, W. L. (1977) Low incidence of estrogen receptor in breast carcinomas with rapid rates of cellular replication. *Cancer*, **40**, 2290.

Papaioannou, A. N., Tany, F. J. & Folk, H. (1967) Fate of patients with recurrent carcinoma of the breast. *Cancer*, **20**, 371.

Roberts, M. M., Rubens, R. D., King, R. J. B. & 4 others (1978) Oestrogen receptors and the response to endocrine therapy in advanced breast cancer. *Br. J. Cancer*, **38**, 431.

Shimkin, M. B., Lucia, E. L., Low-Beer, B. V. A. & Bell, H. G. (1954) Recurrent cancer of the breast. *Cancer*, **7**, 29.

Stewart, J. F., King, R. J. B., Sexton, S. A., Millis, R. R., Rubens, R. D. & Hayward, J. L. (1981) Oestrogen receptors, sites of metastatic disease and survival in recurrence breast cancer. *Eur. J. Cancer*, **17**, 449.

Taylor, S. G. (1962) Endocrine ablation in disseminated mammary carcinoma. *Surg. Gynecol. Obstet.*, **115**, 443.

Walt, A. J., Singlakowinta, A., Brooks, S. C. & Cortez, A. (1976) The survival implications of estrophile protein estimations in carcinoma of the breast. *Surgery*, **80**, 506.